# A Benchmark Side-by-Side Comparison of Two Well-Established Protocols for *in vitro* Hematopoietic Differentiation From Human Pluripotent Stem Cells

**DOI:** 10.3389/fcell.2021.636704

**Published:** 2021-05-21

**Authors:** Francisco Gutierrez-Agüera, Virginia Rodriguez-Cortez, Paolo Petazzi, Clara Bueno, Pablo Menendez

**Affiliations:** ^1^Josep Carreras Research Institute, Barcelona, Spain; ^2^Centro de Investigación Biomédica en Red de Cáncer (CIBER-ONC), Instituto de Salud Carlos III (ISCIII), Barcelona, Spain; ^3^Instituciò Catalana de Recerca i Estudis Avançats (ICREA), Barcelona, Spain

**Keywords:** HPSC, hematopoiesis, differentiation, cytokines, WNT/B-CATENIN

## Abstract

The generation of transplantable hematopoietic stem cells (HSCs) from human pluripotent stem cells (hPSCs) remains challenging. Current differentiation protocols from hPSCs generate mostly hematopoietic progenitors of the primitive HSC-independent program, and it remains unclear what is the best combination of cytokines and hematopoietic growth factors (HGFs) for obtaining functional hematopoietic cells *in vitro*. Here, we have used the AND1 and H9 hESC lines and the H9:dual-reporter *RUNX1C*-GFP-*SOX17*-Cherry to compare the hematopoietic differentiation *in vitro* based on the treatment of embryoid bodies (EBs) with the ventral mesoderm inducer BMP4 plus HGFs in the absence (protocol 1) or presence (protocol 2) of stage-specific activation of Wnt/β-catenin and inhibition of Activin/Nodal. Despite a slight trend in favor of protocol 1, no statistically significant differences were observed between protocols at any time point analyzed throughout EB development regarding the frequency of hemogenic endothelial (HE) precursors; CD43+ CD45−, CD45+, and CD45 + CD34 + hematopoietic derivatives; or the output of clonogenic progenitors. Similarly, the kinetics of emergence throughout EB development of both *SOX17* + HE and *RUNX1C* + definitive hematopoiesis was very similar for both protocols. The expression of the early master mesendodermal transcription factors Brachyury, MIXL1, and KDR revealed similar gene expression kinetics prior to the emergence of *RUNX1C* + definitive hematopoiesis for both protocols. Collectively, the simpler protocol 1 is, at least, as efficient as protocol 2, suggesting that supplementation with additional morphogens/HGFs and modulation of Activin/Nodal and Wnt/β-catenin pathways seem dispensable for *in vitro* hematopoietic differentiation of hPSCs.

## Introducton

Directed differentiation of human pluripotent stem cells (hPSCs) into specific cell types would enable the generation of large numbers of patient- or donor-derived cells for regenerative medicine and the implementation of unique *in vitro* models for studying developmental biology, disease modeling, and drug screening (Menendez et al., 2006). In the hematopoietic setting, the generation of transplantable hematopoietic stem cells (HSCs) from hPSCs remains challenging because both the primitive and definitive developmental programs are intermingled, and current hPSC differentiation protocols generate mostly hematopoietic progenitors of the primitive HSC-independent program (Medvinsky et al., 2011). However, multiple studies have reported the generation of distinct hematopoietic cell types from hPSCs *in vitro*, either by co-culturing them with stromal cell layers such as OP9 cells or by directing their differentiation through embryoid body (EB) development with specific morphogens and hematopoietic growth factors (HGFs) (Demirci et al., 2020).

Hematopoietic development from hPSCs arises from early VEGF receptor (KDR) + CD34−CD31− mesodermal progenitors, and it transitions through CD34 + CD31 + CD45− hemogenic endothelium (HE) precursors (Chadwick et al., 2003; Wang et al., 2004). The expression of glycophorin A (CD235) is used as a surrogate marker to identify the primitive (CD235+) or definitive (CD235−) hematopoietic potential (Sturgeon et al., 2014). HE precursors further differentiate toward CD43 + CD45− and then CD45 + hematopoietic cells (Chadwick et al., 2003; Menendez et al., 2004; Wang et al., 2004, 2005). Early protocols for the successful EB-based *in vitro-*directed differentiation of hPSCs into hematopoietic cells relied on the use of the master early ventral mesoderm inducer BMP4 and different cocktails of HGFs including the early acting hematopoietic cytokines SCF and FLT3L (Chadwick et al., 2003; Wang et al., 2004, 2005; Ledran et al., 2008; Ditadi et al., 2017). Further studies over the last decade suggested that Activin/Nodal and Wnt/β-catenin pathways regulate primitive vs. definitive *in vitro* hematopoietic specification from hPSCs (Sturgeon et al., 2014; Ditadi and Sturgeon, 2016; Ditadi et al., 2017). These studies suggest that the specification of definitive hematopoiesis requires early stage-specific activation of Wnt/β-catenin and inhibition of Activin/Nodal signaling pathways, which is efficiently achieved by treatment with the GSK-3 inhibitor CHIR99021, a Wnt agonist, and the Activin/Nodal inhibitor SB-431542, respectively (Bendall et al., 2007; Kennedy et al., 2012). Although many studies have investigated early hematopoietic development by interrogating the role of instructive transcription factors, it remains unclear what is the best combination of morphogens, cytokines, and HGFs to be used for obtaining functional hematopoietic cells *in vitro*. Here, we have compared the hematopoietic differentiation *in vitro* of two well-established protocols which rely on EB treatment with BMP4 plus a different cocktail HGFs in the absence or presence of stage-specific activation of Wnt/β-catenin and inhibition of Activin/Nodal.

## Materials and Methods

### Maintenance of hPSC Lines

Human embryonic stem cell (hESC) lines, including the dual reporter *SOX17*^*mCHERRY/W*^ *RUNX1C*^*GFP/W*^ H9 cells [kindly provided by Prof. Andrew Elefanty (Murdoch Children’s Research Institute, Monash University, VIC, Australia) and Dr. Andrea Ditadi (Ospedale San Raffaelo, Milan, Italy)], were maintained undifferentiated in T25 flasks on a layer of irradiated murine embryonic fibroblasts in complete Dulbecco’s modified Eagle’s medium (DMEM) containing 20% knockout (KO) serum replacement and 8 ng/ml basic fibroblast growth factor (bFGF) as extensively described (Chadwick et al., 2003; Ramos-Mejia et al., 2014; Bueno et al., 2019). The medium was changed daily and cells were passaged weekly by dissociation with 1:1 collagenase type IV:dispase (Invitrogen, Carlsbad, CA, United States). Cultures were visualized daily by phase contrast microscopy. Approval for the hESC work was obtained from our local health authorities and the Spanish National Pluripotent Ethical Committee (0336E/14973/2017).

### Hematopoietic Differentiation From hPSCs by EB Formation

**FIGURE 1:**
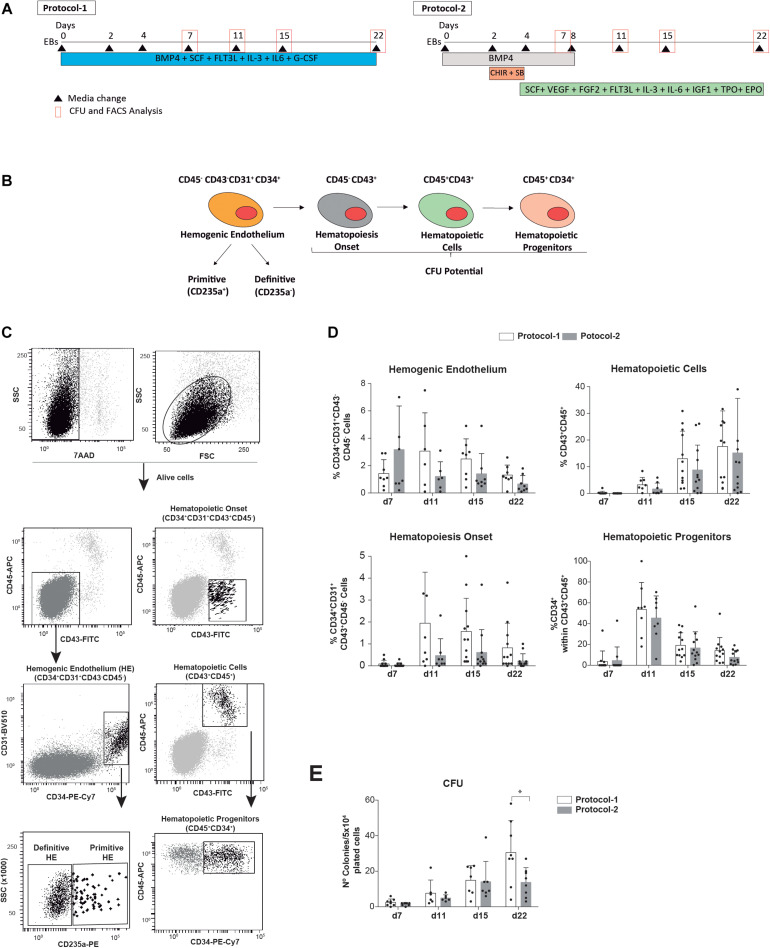
Side-by-side comparison of two well-established EB-based directed hematopoietic differentiation protocols. **(A)** Schematic of the differentiation protocols (protocol 1 and protocol 2) and time-point analyses. **(B)** Cartoon depicting the hematopoietic cell fate specification through the formation of hEBs. **(C)** Representative FACS analysis and identification of the indicated alive hematopoietic cell populations analyzed during hematopoietic commitment of hPSCs. **(D)** Frequency of alive (7AAD–) HE precursors (CD45–CD43–CD34 + CD31 +), CD43 + CD45– and CD45 + hematopoietic cells, and hematopoietic progenitors (CD45 + CD34 +) analyzed at the indicated time points throughout EB development (*n* = 8). **(E)** Clonogenic progenitors detected by hematopoietic CFU assays were analyzed at the indicated time points (*n* = 8). Each individual dot represents the value for each independent *in vitro* differentiation experiment. Data were plotted as mean ± SD. n.s., not significant. ^*^p value < 0.05.

On the day of passage, undifferentiated hESCs at confluence in T25 culture flasks (∼8 × 10^6^ alive cells) were first treated with collagenase type IV:dispase for 1 h at 37°C, and dispersed cells were transferred to six-well low-attachment plates (∼1 × 10^6^ alive cells/well/condition; alive cells were measured by trypan blue exclusion) and incubated overnight in differentiation medium (DM; KO-DMEM supplemented with 20% fetal bovine serum, 1% non-essential amino acids, 1 mmol/L L-glutamine, and 0.1 mmol/L β-mercaptoethanol). Media changes and supplementation with BMP4, different HGFs, and inhibitors were performed as in Figure 1A. Concentrations used were as follows: 3 μM CHIR99021, 3 μM SB-431542, 25 ng/ml BMP4, 300 ng/ml stem cell factor (SCF), 300 ng/ml FMS-like tyrosine kinase 3 ligand (Flt3L), 10 ng/ml interleukin (IL)-3, 10 ng/ml IL-6, 50 ng/ml granulocyte-colony stimulating factor (G-CSF), 15 ng/ml VEGF, 10 ng/ml basic fibroblast growth factor 2 (FGF2), 25 ng/ml insulin-like growth factor-1 (IGF1), 30 ng/ml thrombopoietin (TPO), and two IU erythropoietin (EPO) (all from R&D Systems, Minneapolis, MN, United States) (Chadwick et al., 2003; Sturgeon et al., 2014; Ditadi and Sturgeon, 2016). In the serum-free experiments, the basal media SFD composed of IMDM and Ham’s F12 (Gibco) supplemented with L-glutamine (2 mM), ascorbic acid (1 mM), monothioglycerol (MTG, 4 × 10^–4^ M; Sigma), transferrin (150 μg/ml), N2 (100×), B27 (100×), and bovine serum albumin (BSA) 0.1% was used.

Embryoid bodies were dissociated at different time points during development using collagenase B and enzyme-free cell dissociation buffer (Invitrogen). Dissociated cells were stained with anti-CD34-PE, anti-CD31-FITC, anti-CD45-APC or anti-CD34-PE-Cy7, CD31-BV510, anti-glycophorin A-PE, anti-CD43-FITC, and anti-CD45-APC antibodies and 7-actinomycin D (7AAD) for exclusion of dead cells and analyzed using a FACS Canto flow cytometer (BD Biosciences). The emergence of alive (7AAD−) *SOX17* + and *RUNX1C* + cells during EB development was analyzed using Cherry and GFP reporters, respectively. Colony-forming unit (CFU) assays were performed at different time points along EB differentiation by plating 5 × 10^4^ EB-derived cells onto serum-free methylcellulose H4435 (Stem Cell Technologies, Vancouver, BC, Canada). Colonies were scored after 12 days using standard CFU scoring assays (Chadwick et al., 2003; Ramos-Mejia et al., 2014; Bueno et al., 2019).

### Real-Time Reverse Transcriptase-Polymerase Chain Reaction

DNAse-treated total RNA was extracted from EBs using the Maxwell RSC simplyRNA Cells Kit (Promega, Madison, WI, United States). Two hundred and fifty nanograms of RNA was retrotranscribed with random hexamers using the Superscript III first-strand synthesis kit (Thermo Fisher Scientific, Waltham, MA, United States). The resulting cDNA was diluted 1:2 and analyzed for differential gene expression using the PowerUp SYBR Green Master Mix (Thermo Fisher Scientific, Waltham, MA, United States) on a Bio-Rad CFX384 qPCR Platform (Bio-Rad, Hercules, CA, United States). Relative expression of the mesoderm markers was calculated with the ΔΔCt method using *RPL19* as the housekeeping gene. Primer sequences were as follows: *KDR*: Fw-CCACTGGTATTGGCAGTTGGA, Rev-CACAAGGGTATGGGTTTGTCAACT (80 bp, exons 10–11); *T/BRACHYURY*: Fw-ATGAGCCTCGAATCCACATAGT, Rev-TCCTCGTTCTGATAAGCAGTCA (108 bp, exon 3); *MIXL1*: Fw-GGATCCAGGTATGGTTCCAG, Rev-GGAGCACAGTGGTTGAGGAT (130 bp, exons 1–2); and *RPL19*: Fw-GCGGAAGGGTACAGCCAAT, Rev-AGCAGCCGGCGCAAAATCC (78 bp, exon 4). The absence of genomic DNA contamination was confirmed by running RT controls.

### Statistical Analyses

Data are plotted as mean ± standard deviation (SD). *p* values were calculated using paired two-tailed Student’s *t* test for each time point using the Prism software version 8.0 (GraphPad Prism Software Inc., San Diego, CA, United States).

## Results

We have compared whether two extensively employed EB-based directed differentiation protocols influence hPSC-derived hematopoietic differentiation *in vitro*. The differentiation protocols exclusively differed in the cocktail of morphogens and HGFs used during EB development (Figure 1A). The simpler protocol was developed by Bhatia’s lab in 2003 and used the ventral mesoderm inducer BMP4 plus the HGFs SCF, FLT3L, IL-3, IL-6, and G-CSF throughout the entire 22-day differentiation protocol (Chadwick et al., 2003; Menendez et al., 2004; Wang et al., 2004, 2005) (termed as protocol 1, Figure 1A). The other differentiation protocol was developed later on by Keller’s lab and employed a more complex setup of morphogens and HGFs suggested to more efficiently promote EB differentiation toward the definitive hematopoietic program (Kennedy et al., 2012; Ditadi and Sturgeon, 2016; Ditadi et al., 2017). It includes an 8-day treatment with BMP4 and a concomitant stage-specific (day 2 to day 4 of EB development) Wnt/β-catenin activation with the GSK-3 inhibitor CHIR99021 and Activin/Nodal inhibition with the inhibitor SB-431542 followed by treatment with SCF, VEGF, FGF2, FLT3L, IL-3, IL-6, IGF1, TPO, and EPO from day 4 of EB development onward (termed as protocol 2, Figure 1A). The *in vitro* efficiency of both protocol 1 and protocol 2 was compared side-by-side using the hESC lines AND1 and H9 (*n* = 8 independent experiments). The frequency of HE precursors (CD45−CD43−CD34 + CD31+), hematopoietic cells (both CD43 + CD45− and CD43 + CD45 +), and hematopoietic progenitors (CD45 + CD34 +) as well as the output of clonogenic progenitors detected by hematopoietic CFU assays was analyzed at indicated time points throughout EB development (Figures 1B–E). Overall, although a slight trend in favor of protocol 1 was consistently observed, no statistically significant differences were observed in the frequency of either HE precursors; CD43 + CD45−, CD45+, or CD45 + CD34 + hematopoietic derivatives (Figure 1D); or the output of clonogenic progenitors (Figure 1E) at any time point analyzed (from day 7 to day 22 of EB development). Of note, ∼95% of the HE precursors were CD235− regardless of the protocol employed (Figure 1C).

**FIGURE 2:**
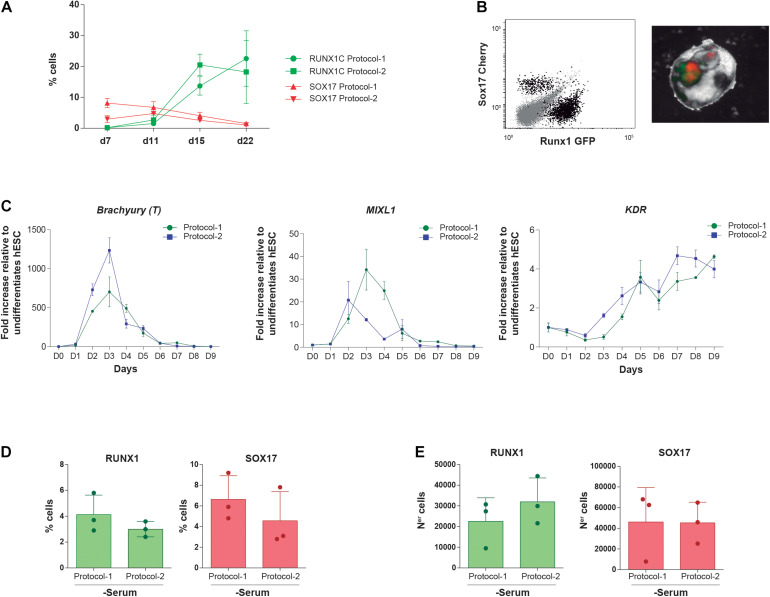
Impact of both differentiation protocols on the emergence kinetics of *SOX17* + HE and *RUNX1C* + definitive hematopoietic cells. **(A)** Frequency of alive (7AAD–) *SOX17*:Cherry + and RUNX1C:GFP + cells along EB differentiation with protocol 1 and protocol 2 (*n* = 4). **(B)** *Left panel*, representative flow cytometry plots displaying how (SOX17/Cherry +) and definitive hematopoietic cells (RUNX1/GFP +) are identified. The right panel shows mCherry and GFP expression within differentiating EBs by fluorescence microscopy. **(C)** Gene expression kinetics of the mesendodermal transcription factors Brachyury (T), MIXL1, and KDR during mesoderm induction and early hematopoietic differentiation with protocol 1 and protocol 2 (*n* = 4). **(D,E)** Frequency **(D)** and absolute numbers **(E)** of RUNX1/GFP + definitive hematopoietic cells and SOX17/Cherry + HE cells at endpoint (day 22) of *in vitro* differentiation using protocol 1 or protocol 2 in a serum-free media. Data were plotted as mean ± SD. n.s., not significant.

We next took advantage of the *RUNX1C*-GFP-*SOX17*-Cherry dual reporter H9 hESC line to track the endothelial (*SOX17* +) to definitive hematopoietic (*RUNX1C*+) transition (Ng et al., 2016). Our data showed that the protocol employed (protocol 1 vs protocol 2) had no impact on the kinetics of emergence of either *SOX17* + HE or *RUNX1C* + definitive hematopoiesis throughout the 22 days of EB development (*n* = 4, Figures 2A,B). The limited impact of the early stage-specific activation of Wnt/β-catenin and inhibition of Activin/Nodal pathways in definitive hematopoietic development *in vitro* was further confirmed at the molecular level. Real-time reverse transcriptase-PCR-based expression of the early master mesendodermal transcription factors Brachyury, MIXL1, and KDR revealed very similar gene expression kinetics prior to the emergence of *RUNX1C* + definitive hematopoiesis (*n* = 4, Figure 2C).

Protocol 1 has been historically used in the presence of serum and protocol 2 in the absence of serum. To assess whether the presence of serum masks the differences between both protocols, the *RUNX1C*-GFP-*SOX17*-Cherry H9 hESC line was differentiated (*n* = 2) for 22 days in serum-free medium with the morphogens and HGFs of protocol 1 vs. protocol 2. As shown in Figures 2D–E, no significant differences were observed either in frequencies or in absolute numbers between protocols for either *RUNX1C* + or *SOX17* + cells, indicating that the lack of significant differences in the hematopoietic output between both protocols is not attributable to (masked by) the presence of serum. Collectively, our *in vitro* data using different hESC lines demonstrate that the simpler protocol 1 is, at least, as efficient as protocol 2, suggesting that the supplementation with additional morphogens/HGFs and modulation of Activin/Nodal and Wnt/β-catenin pathways seem dispensable for *in vitro* hematopoietic differentiation of hPSCs.

## Discussion

Human PSCs have long been postulated as an unprecedented model for studies on human development and disease modeling and for drug testing (Menendez et al., 2006). Many studies have investigated early hematopoietic development by interrogating the role of instructive transcription factors (Real et al., 2012, 2013; Ramos-Mejia et al., 2014; Ayllón et al., 2015; Bueno et al., 2019). Nonetheless, it remains unclear regarding the best combination of cytokines and growth factors, the timing of treatment, and the methodology to be used for obtaining fully functional hematopoietic cells *in vitro*. There are a large number of published *in vitro* protocols for differentiation of hPSCs toward hematopoietic cells. These protocols largely differ in many aspects including (i) the use of several EB systems (spin and non-spin EBs) vs. stromal cells such as OP9, MSCs, AGM feeders, fetal liver feeders, etc.; (ii) the absence or presence of sera from different mammalian sources; and (iii) a never-ending combination of morphogens and HGFs, among others. However, none of the protocols used so far render hPSC hematopoietic derivatives capable of reconstituting the hematopoietic system in immune-deficient mice.

Two well-established, widely used *in vitro* hematopoietic differentiation protocols exist. The first protocol, initially developed by Bhatia’s lab (termed as protocol 1 in this study), relies on EB treatment with the ventral mesoderm inducer BMP4 together with the HGFs SCF, FLT3L, IL-3, IL-6, and G-CSF for further blood specification for 22 days (Chadwick et al., 2003; Menendez et al., 2004; Wang et al., 2004, 2005). The other protocol developed by Keller’s lab employs BMP4 together with a concomitant stage-specific (days 2–4 of EB development) Wnt/β-catenin activation with the GSK-3 inhibitor CHIR99021 and Activin/Nodal inhibition with the inhibitor SB-431542 followed by treatment with SCF, VEGF, FGF2, FLT3L, IL-3, IL-6, IGF1, TPO, and EPO from day 4 of EB development onward (termed as protocol 2 in this study) (Kennedy et al., 2012; Sturgeon et al., 2014; Ditadi and Sturgeon, 2016; Ditadi et al., 2017). The GSK-3 inhibitor CHIR99021 and the Activin/Nodal inhibitor SB-431542 were selected based on previous reports showing that WNT3A exposure during mesoderm patterning of hESCs suppresses primitive hematopoiesis (Gertow et al., 2013) and that WNT agonists or ACTIVIN antagonists may support definitive hematopoiesis from hPSCs (Kennedy et al., 2012; Sturgeon et al., 2014). Both inhibitors are used between day 2 and day 4 of EB differentiation, the period during which HOX gene expression is initiated (Ramos-Mejia et al., 2014; Ng et al., 2016).

In this study, we have used several wild-type and reporter hPSC lines to compare the hematopoietic differentiation *in vitro* based on the treatment of EBs with BMP4 plus HGFs in the absence (protocol 1) or presence (protocol 2) of stage-specific activation of Wnt/β-catenin and inhibition of Activin/Nodal. We demonstrate that the simpler protocol 1 is, at least, as efficient as protocol 2, suggesting that the supplementation with additional morphogens/HGFs and modulation of Activin/Nodal and Wnt/β-catenin pathways seem dispensable for yielding a higher number of hematopoietic (progenitor) derivatives for subsequent downstream *in vitro* applications. Furthermore, preliminary data suggest that a similar trend of hematopoietic differentiation was observed in serum-free conditions indicating that the lack of significant differences in the hematopoietic output between both protocols is not masked by the presence of serum. Further side-by-side *in vitro* comparisons should comprehensively investigate the hematopoietic output of the multiple combinations of morphogens and HGFs used in parallel or sequentially in EB-based hematopoietic differentiation protocols reported from multiple laboratories.

## Data Availability Statement

The raw data supporting the conclusions of this article will be made available by the authors, without undue reservation.

## Ethics Statement

Approval for hESC work was obtained from the ISCIII-Comisión Nacional de Garantías (0336E/14973/2017).

## Author Contributions

FG-A designed and performed the experiments and analyzed the data. VR-C and PP performed the experiments. CB and PM conceived the study, designed the experiments, analyzed the data, and wrote the manuscript. All authors contributed to the article and approved the submitted version.

## Conflict of Interest

The authors declare that the research was conducted in the absence of any commercial or financial relationships that could be construed as a potential conflict of interest.
